# Comparative features of infections of two Massachusetts (Mass) infectious bronchitis virus (IBV) variants isolated from Western Canadian layer flocks

**DOI:** 10.1186/s12917-018-1720-9

**Published:** 2018-12-10

**Authors:** Aruna Amarasinghe, Upasama De Silva Senapathi, Mohamed Sarjoon Abdul-Cader, Shelly Popowich, Frank Marshall, Susan C. Cork, Frank van der Meer, Susantha Gomis, Mohamed Faizal Abdul-Careem

**Affiliations:** 1 Department of Ecosystem and Public Health, Faculty of Veterinary Medicine, University of Calgary, Health Research Innovation Center 2C53, 3330 Hospital Drive NW, Calgary, AB T2N 4N1 Canada; 2 Department of Veterinary Pathology, Western College of Veterinary Medicine, University of Saskatchewan, Saskatoon, SK S7N 5B5 Canada; 3 Marshall Swine and Poultry Health Services, 3831- Bay G- 44 Ave, Camrose, AB T4V 3T1 Canada

**Keywords:** Infectious bronchitis virus, Whole genome sequencing, Tissue distribution, Pathogenicity, Macrophage response

## Abstract

**Background:**

Infectious bronchitis virus (IBV) is one of the leading causes of mortality and morbidity in chickens. There are numerous serotypes and variants, which do not confer cross protection resulting in failure of currently used IBV vaccines. Although variant IBV isolates with major genetic differences have been subjected to comparative studies, it is unknown whether minor genetic differences in IBV variants within a serotype are different in terms of pathogenesis and eliciting host responses. Two Massachusetts (Mass) variant IBV isolates recovered from commercial layer flocks in the Western Canadian provinces of Alberta (AB) and Saskatchewan (SK) were compared genetically and evaluated for their pathogenicity, tissue distribution and ability to recruit and replicate in macrophages.

**Results:**

Although whole genome sequencing of these two Mass IBV isolates showed low similarity with the M41 vaccinal strain, they had an identical nucleotide sequence at open reading frames (ORFs) 3a, 3b, envelop (E), matrix (M), 5a and 5b. The rest of the ORFs of these 2 IBV isolates showed 99.9% nucleotide similarity. However, upon experimental infection, we found that the IBV isolate originating from AB was different to the one that originated in SK due to higher tracheal lesion scores and lower lung viral replication and lower genome loads in cecal tonsils. Nevertheless, both IBV isolates elicited host responses characterized by significant macrophage recruitment to the respiratory tract and there was evidence that both IBV isolates replicated within tracheal and lung macrophages.

**Conclusions:**

Overall, this study shows that Mass variant IBV isolates, although possessing minor genetic variations, can lead to significant differences in pathogenicity in young chickens. Further studies are required to investigate the pathogenicity of these two Mass variant IBV isolates in laying hens.

**Electronic supplementary material:**

The online version of this article (10.1186/s12917-018-1720-9) contains supplementary material, which is available to authorized users.

## Background

Infectious bronchitis virus (IBV) belongs to the family *Coronaviridae*. Traditionally, IBV is considered to be a host-specific respiratory pathogen in chickens and IBV initially replicates at the route of entry, the tracheal mucosa [1, 2]. However, identification of new variants and/or serotypes of IBV have shown a wide variation of tissue tropism including urinary [3–10], gastrointestinal [6, 9, 11, 12], oviduct [9, 13] and bursa of Fabricius [11, 14]. IBV is known to replicate in the reproductive tract epithelium in layers leading to reduced egg production and shell defective eggs [15, 16]. False layer syndrome, which is associated with cystic oviduct formation occurs with IBV infection in early life [17, 18]. IBV can also replicate in the testes of cockerels [19].

An array of serotypes and strains of IBV infect chickens throughout the world [2]. Genetic events such as insertion(s) and deletions [20, 21], point mutations [22], and recombination [23–27] contribute to genomic variations of IBV [28]. The spike 1 (S1) gene is highly variable among IBV strains and it encodes epitopes, which bind to neutralizing antibodies [29]. A change in the amino acid sequence as small as 2 to 3% in the S1 subunit can result in changes in the antigenicity of the virus [30]. Based on these changes of the S1 protein, numerous IBV strains have been characterized throughout the world [1]. Therefore, either the partial [31–33] or the full-length [34–39] of the S1 glycoprotein gene has been used in the molecular characterization of IBV isolates. However, using whole genome sequencing it has been observed that genes other than S1 may play a role in the pathogenicity of IBV infection [40, 41]. Currently, there are no IBV reference full genome sequences available for Canadian IBV isolates but partial [33] or complete [37] S1 sequences are available.

Previously, comparative studies have been conducted to elucidate differential pathogenicity and host responses between variant IBV isolates with larger genome variability such as nephropathogenic and Massachusetts (Mass) IBV isolates recovered from various geographical areas [18, 42–46]. Since it is well known that very minor changes in the genome of viruses [47] including IBV [48, 49] can lead to difference in pathogenicity, comparative studies involving variant IBV isolates are required.

A wide array of IBV variants are affecting commercial broiler, layer and breeder flocks in Canada [33, 37, 50, 51]. For example, Mass type IBV variants are impacting the commercial egg production in Western Canada [50]. This study showed that Mass type IBV infection of 27-week old layers at peak production, lead to loss of egg production for about 2 weeks followed by production of shell less, small and defective eggs for another 2 days before the egg production bounced back to normal production. The duration of the loss of marketable eggs appears to be 16 days. In an 8000 bird layer flock of Western Canada, we observed 47.6% drop in egg production for 10 days and we isolated a Mass type variant IBV from this flock. Based on Egg Farmers of Alberta’s price of $2.15/dozen of large eggs, the loss for 10 days is calculated to be $6823 for this particular outbreak and the loss will be higher if the infection persists beyond 10 days or reoccur. In addition to this direct loss of egg production, egg grading stations need to outsource supply of eggs during these production drops and also some of the affected birds may succumb to secondary bacterial infections without a recovery, hence the indirect losses are substantially higher than the direct costs of IBV infection.

The present study was conducted with the aim to elucidate the genetic differences of two Mass variant IBV isolates (15AB-01 and 15SK-02) that originated from layer flocks in Western Canada, Alberta (AB) and Saskatchewan (SK). Additionally, we aimed to describe whether these genetic changes in Mass isolates 15AB-01 and 15SK-02 lead to differential pathogenicity, tissue distribution and macrophage responses in the respiratory tract.

## Results

### Molecular characterization of the mass IBV isolates, 15AB-01 and 15SK-02

For the isolate 15AB-01, total of 525,998 and 908,860 read sets were obtained from the direct allantoic fluid sample and layered virus sample, respectively from the Illumina MiSeq workflow. From these, a total of 418,446 and 825,147 reads were aligned with the reference IBV genomes. For isolate 15SK-02, a total of 4,355,902 and 3,731,578 reads were obtained from the direct allantoic fluid sample and layered virus sample, respectively, of which, a total of 3,146,584 and 3,152,622 reads were aligned with the IBV reference genomes. The number of reads obtained from Illumina MiSeq for 15SK-02 was 10 times higher than for 15AB-01 and the viral genome concentration of the submitted complimentary (c)DNA was also much higher for 15SK-02 than for 15AB-01 (real-time PCR cycle threshold or Ct value difference of 3.54). Entirely based on the NGS output, the complete genomes obtained for the Mass IBV isolates, 15AB-01 and 15SK-02 were 27.6 kb in length. As determined by the open reading frames (ORF) predictor, both isolates had similar genome organization with 11 ORFs and they were 5′-1a-1b-S-3a-3b-E-M-5a-5b-N-3′ (Table 1). Between the isolates, 15AB-01 and 15SK-02, there was 99.9% nucleotide similarity. With the M41 vaccine strain, 15AB-01 and 15SK-02 shared a genetic similarity of 92.8 and 92.7%, respectively. 15AB-01 and 15SK-02 Mass IBV isolates shared 92.8 and 92.7% nucleotide similarity with the M41 vaccine strain (GQ504725), respectively. Nucleotide similarities between Mass IBV isolates, 15AB-01 and 15SK-02 across the ORFs 3a, 3b, E, M, 5a and 5b were 100%. The remainder of genes were over 99% identical (Table 2). Nucleotide similarities between Mass IBV isolates, 15AB-01 and 15SK-02 across the hypervariable S gene regions, S1 and S2, respectively were 99.6 and 99.8%. The lowest nucleotide similarity these two isolates shared with the vaccine M41 strain was 86.4% in the ORF 5a and the highest similarity was 100% in the ORF M. The S1 and S2 identities of these two IBV isolates with the M41 vaccine strain were 95.5 and 97.6%, respectively (Table 2). Additionally, our IBV isolates, 15AB-01 and 15SK-02 were compared with important reference strains in Mass type cluster (Table 3) and they are most variable from the Beaudette IBV strain and very closely related to other Mass type IBV strains.

**Table 1:** Genes, coding regions, and deduced proteins of the current Mass IBV isolates, 15AB-01 and 15SK-02

Gene	Translation Frame	Gene location	Nucleotide length (bp)	Number of amino acids
1a	1	529–12,390	11,862	3953
1b	3	12,465–20,423	7959	2652
S	1	20,374–23,862	3489	1162
3a	3	23,862–24,035	174	57
3b	2	24,035–24,229	195	64
E	3	24,210–24,539	330	109
M	1	24,511–25,188	678	225
5a	3	25,539–25,736	198	65
5b	2	25,733–25,981	249	82
N	1	25,924–27,153	1230	409

**Table 2:** Nucleotide similarity of 15AB-01 and 15SK-02 across various genes with M41 vaccine strain GQ504725

Strain	ORF1a	ORF 1b	S1	S2	ORF3a	ORF3b	E	M	ORF5a	ORF5b	N
15AB-01	90.7	96.8	95.5	97.6	91.2	96.9	94.5	100	86.4	89.2	94.1
15SK-02	90.6	96.8	95.5	97.9	91.2	96.9	94.5	100	86.4	89.2	94.5
^a^15AB-01 Vs 15SK-02	99.9	99.9	99.6	99.8	100	100	100	100	100	100	99.5

^a^Third raw depicts the nucleotide similarities between current Mass IBV isolates 15AB-01 and 15SK-02 across various genes

**Table 3:** Nucleotide identities of 15AB-01 and 15SK-02 across various genes with Mass-type reference strains

Strain	ORF 1a	ORF 1b	S1	S2	ORF 3a	ORF 3b	E	M	ORF 5a	ORF 5b	N
Beaudette	91.5/91.5	99.9/93.9	97.8/97.8	98.1/98.2	97.7/97.1	98.2/98.2	98.7/98.7	98.8/98.8	98.9/98.9	90.8/90.8	92.6/92.7
TN92–03 (India)	93.2/93.2	94.0/93.9	97.2/97.0	98.3/98.4	97.7/98.3	97.0/97.0	98.4/98.4	99.0/99.0	100/100	95.6/95.6	97.6/99.8
Mass41/1972 (USA)	99.7/99.7	99.5/99.5	99.8/99.8	99.2/99.3	98.8/99.4	100/100	99.7/99.7	99.0/99.0	100/100	100/100	99.6/99.8
Mass/USA (AY851295)	100/99.9	99.9/99.9	99.9/99.9	99.9/99.9	100/99.4	100/100	99.7/99.7	100/100	100/100	100/100	99.8/99.6
ck/CH/LHLJ/091205 (China)	99.9/99.9	99.9/99.9	99.9/99.9	99.9/99.9	100/99.4	100/100	100/100	100/100	100/100	100/100	100/99.8
M41/USA (DQ834384)	100/100	100/99.9	99.9/99.9	99.7/99.7	100/99.4	100/100	100/100	99.9/99.9	100/100	100/100	99.7/99.8
Mass/USA (GQ504724)	100/100	99.9/99.9	99.9/99.9	99.8/99.8	100/99.4	100/100	100/100	100/100	100/100	99.6/99.6	99.8/99.8
M41/1965 (USA)	99.9/99.9	100/100	99.9/99.9	99.8/99.8	98.8/99.4	100/100	100/100	100/100	100/100	100/100	99.6/99.8
M41/1985 (USA)	100/100	99.9/100	99.9/100	99.8/99.8	98.8/99.4	100/100	100/100	100/100	100/100	100/100	99.7/99.8

In each cell of the table, the first value is the nucleotide identity shared with the IBV isolate 15AB-01 and the second value is the nucleotide identity shared with the IBV isolate 15SK-02

**Fig. 1:**
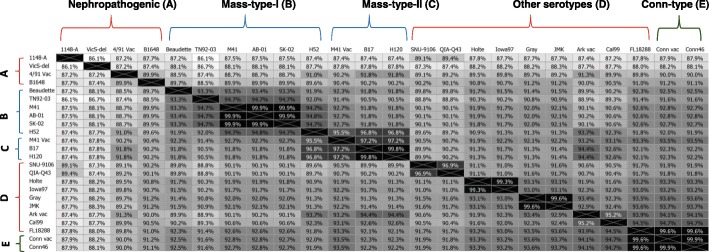
Nucleotide similarity matrix and heat maps of 23-selected reference strains and 2 current Mass IBV isolates. Full genome sequences of Western Canadian isolates (15AB-01 and 15SK-02) were aligned with 23 reference sequences retrieved from the GenBank. The resulting genetic distance matrix along with heat maps based on the similarity index is shown

**Fig. 2:**
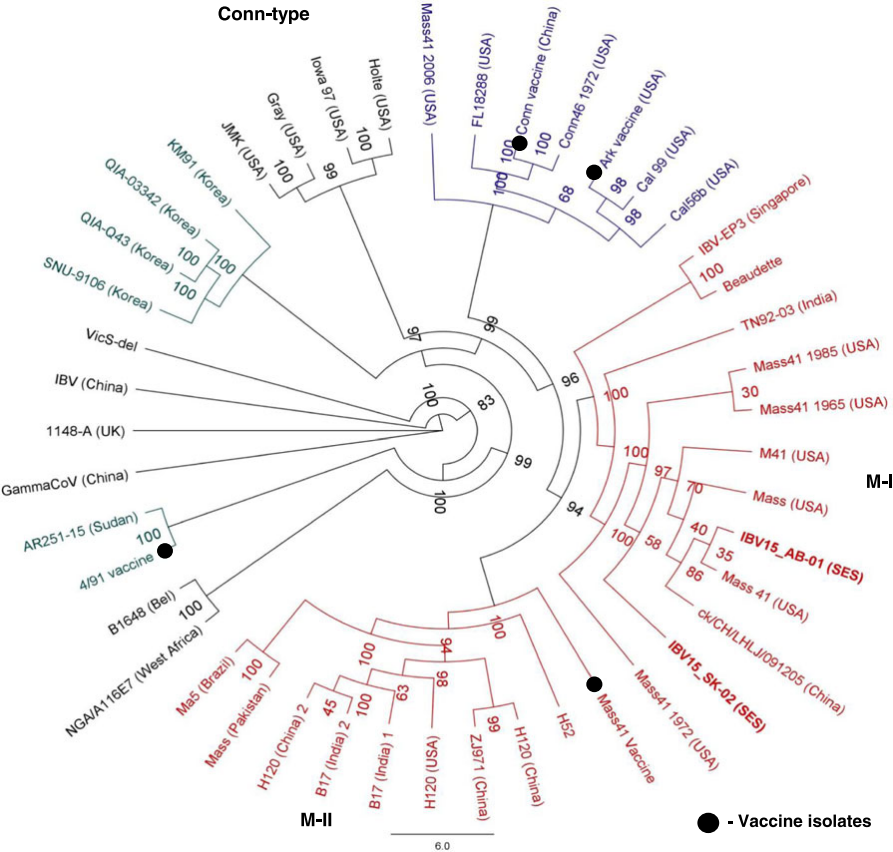
Phylogenetic tree of 45 IBV whole genome sequences including Western Canadian Mass IBV isolates. A maximum likely hood phylogenetic tree was inferred based on the whole genomes of 15AB-01 and 15SK-02 isolates of IBV and 43 IBV genome sequences retrieved from GenBank using RAxML with GTR GAMMA nucleotide substitution model with 1000 bootstrap iterations

Full genome nucleotide distance matrix and heat maps of these isolates with 23 selected reference sequences are shown in the Fig. 1. The highest similarity of 15AB-01 and 15SK-02 shared with M41 strain (USA) was 99.9%. The lowest similarity was 87.5% with the United Kingdom (UK) strain 1148 (Fig. 1). Phylogenetic reconstruction with reference IBV strains based on the full genome sequence revealed a close relation with two IBV isolates to the USA Mass type virus (Fig. 2). The isolate 15AB-01 appeared to be more recently evolved than the 15SK-02 isolate. However, clear separation of these two IBV isolates (cluster M1) from the vaccine M41 strain (Cluster M11) is evident (Fig. 2).

### Clinical observations and body weight changes following IBV infection of chickens

**Fig. 3:**
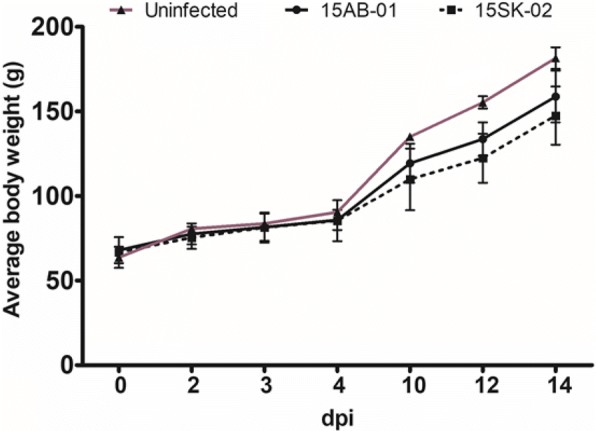
Bodyweight changes following infection with Western Canadian Mass-type IBV isolates. The average body weight of each group (IBV 15AB-01, IBV 15SK-02 and uninfected control) of chickens at different time points are depicted in the graph. The error bars represent the ±standard error of mean (SEM)

From 2 to 8 days post-infection (dpi), there were two birds with mildly increased respiration (more labored and higher frequency) from the IBV (15AB-01) infected group. On 9 and 10 dpi, there were 3 birds with mildly increased breathing. Only one bird continued to show a mild breathing increase by 11 dpi and thereafter all birds were bright and alert. In contrast, 2 birds from the 15SK-02 infected group started showing increased breathing as late as 7 dpi and continued until 11 dpi. The control birds were alert and active throughout. The highest body weight gain was achieved by the uninfected control group followed by the group infected with the isolate 15AB-01 and 15SK-02 (Fig. 3). However, the difference in bodyweights was not significant (*p* > 0.05).

### Histopathological changes following IBV infection of chickens

**Fig. 4:**
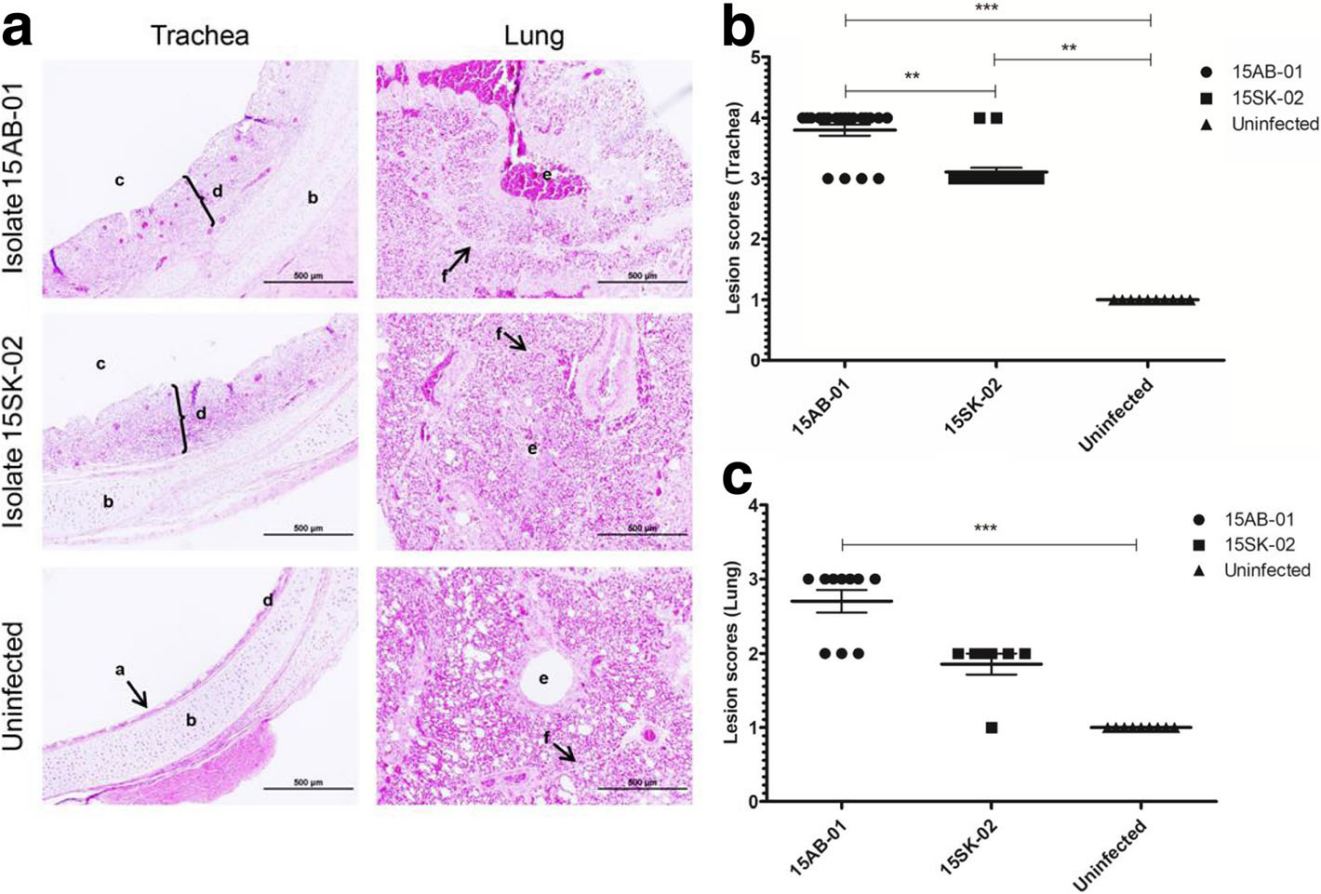
Histological changes in trachea and lungs induced by Western Canadian Mass-type IBV isolates. The representative images of trachea and lungs (**a**) and average lesion scores of trachea (**b**) and lung (**c**) for each group of chickens (IBV 15AB-01, IBV 15SK-02 and uninfected control) at 4 dpi are depicted. a = cilia, b = tracheal cartilage, c = tracheal lumen, d = tracheal mucosa, e = parabronchial lumen and f = inter-parabronchial septum. The error bars represent the ±SEM. Statistical significance: *p < 0.05, **p < 0.01, ***p < 0.001

Histopathological examination of both trachea and lung at 4 dpi showed lesions suggestive of infectious bronchitis. In the trachea, these histological changes included de-ciliation of tracheal mucosal cells, lymphoid infiltration into mucosa and underlying submucosa, and mucosal detachment from the lamina propria (Fig. 4a). Squamous metaplasia of the tracheal mucosa was prominent in trachea of both IBV infected groups (Fig. 4a). Average lesion score of the trachea infected with the isolate 15AB-01 (*p* < 0.001) and 15SK-02 (*p* < 0.01) were significantly higher than that of trachea from uninfected controls (Fig. 4b). Also lesion scores of the trachea from 15AB-01 infected chicken were significantly higher than that of trachea from chicken infected with isolate 15SK-02 (*p* < 0.01) (Fig. 4b). In the lungs, mononuclear cell infiltration and hemorrhage into the parabronchial lumen were observed in the infected lungs compared to the uninfected controls (Fig. 4a). We observed variation in lung lesion scores within groups, yet significant outliers were not detected when the data were subjected to Grubbs test. The lung lesion scores of the lung from 15AB-01 infected chicken were significantly higher than that of the lungs from uninfected controls. However, the lung lesion score between the lung from 15SK-02 infected chicken and the uninfected controls was not statistically significant (*p* > 0.05) (Fig. 4c).

### IBV genome loads in oropharyngeal and cloacal swabs and tissue samples

**Fig. 5:**
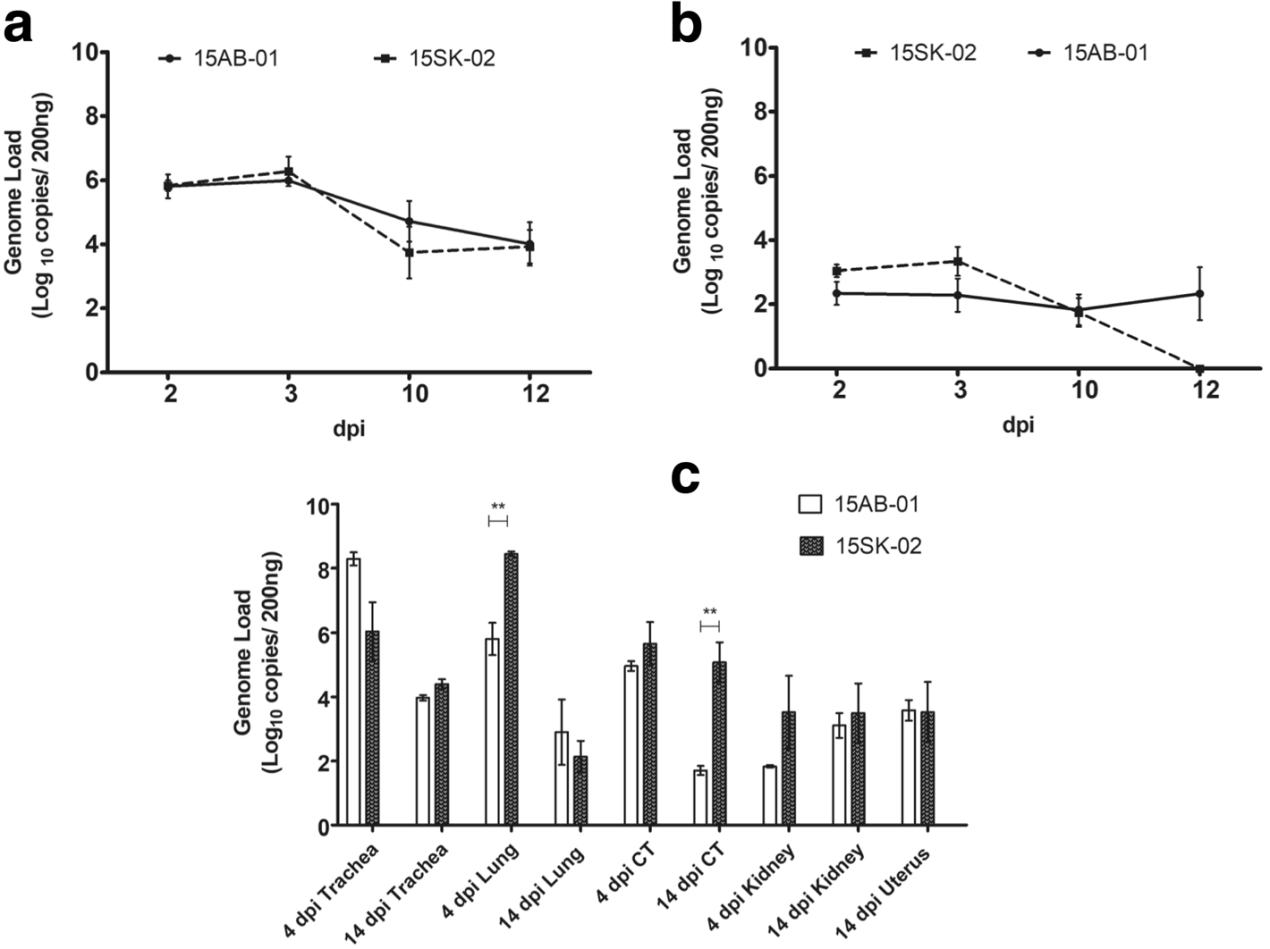
IBV genome loads in swabs and tissues following infection with Western Canadian Mass-type IBV isolates. For each time point, the average log_10_ starting IBV copies per 200 ng of extracted RNA from the oropharyngeal (**a**) and cloacal (**b**) swabs and various tissues (**c**) at 4 and 14 dpi are shown with the SEM. Statistical significance: *p < 0.05, **p < 0.01, ***p < 0.001

The average genome loads on oropharyngeal swabs were higher than the genome loads on cloacal swabs for both Mass IBV isolates at all time points (Fig. 5a and b). In the trachea, a genome load of 1 × 10^6^ up to 3 dpi could be detected in chicken infected with either isolate. By 12 dpi, the genome loads in the trachea declined by 2-folds. The genome load in the cloaca was consistent for the 15AB-01 throughout the observation period. However, in the cloaca of birds infected with 15SK-02 no IBV genome at 12 dpi could be detected (Fig. 5b). No IBV genome load was detected in the uninfected controls (results not shown).

The highest genome loads in tissue were found in trachea and lung tissues of chicken infected with either isolate at 4 dpi, however, the genome load in lung tissue was significantly lower for IBV isolate 15AB-01 (Fig. 5c). At 4 and 14 dpi, the genome loads in trachea were similar for IBV isolates (15AB-01 and 15SK-02). By 14 dpi, tracheal and lung genome loads fell considerably for both isolates. Cecal tonsil genome load was declined by 14 dpi only in birds infected with the isolate 15AB-01 and the difference was statistically significant (*p* < 0.01). There was no difference in genome loads in the kidney tissue between two time points in both strains. The uterine genome loads were available only at 14 dpi for both strains. None of the uninfected control birds had detectable IBV genome loads (results not shown).

### Immunofluorescence data for IBV antigens and macrophages at 4 dpi

**Fig. 6:**
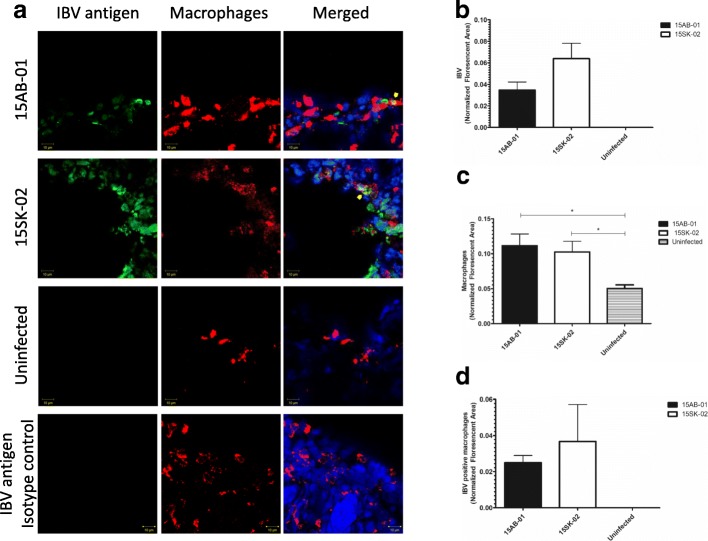
Mass-type IBV isolates recovered from Western Canadian layer flocks increase macrophage numbers in trachea. The representative images of trachea stained for IBV antigens and macrophages (**a**) and quantitative data for IBV antigens (**b**), macrophages (**c**) and IBV antigen positive macrophages (**d**) at 4 dpi are depicted. The error bars represent the ± SEM. Statistical significance: *p < 0.05, **p < 0.01, ***p < 0.001

**Fig. 7:**
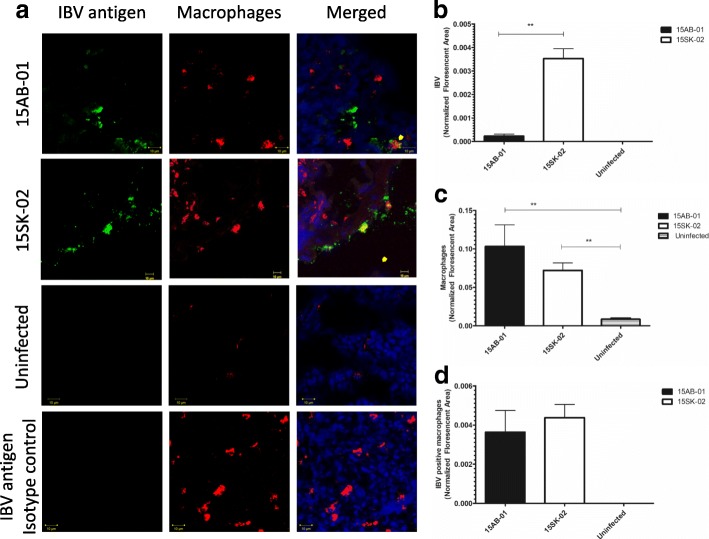
Mass-type IBV isolates recovered from Western Canadian layer flocks increase macrophage numbers in lungs. The representative images of lungs stained for IBV antigens and macrophages (**a**) and quantitative data for IBV antigens (**b**), macrophages (**c**) and IBV antigen positive macrophages (**d**) at 4 dpi are depicted. The error bars represent the ± SEM. Statistical significance: *p < 0.05, **p < 0.01, ***p < 0.001

Similar to IBV genome loads, we did not see a difference in IBV antigen expression in trachea of 15AB-01 and 15SK-02 infected chickens (*p* > 0.05, Fig. 6b). Similar to the IBV genome load, the amount of IBV antigen was significantly lower in 15AB-01 infected lung than that of 15SK-02 infected lung (*p* < 0.01, Fig. 7b). The lung and tracheal macrophage numbers were not significantly different in the two groups of experimentally infected chicken, indicating a similar response to the two strains of Mass IBV used in the study (*p* > 0.05). Macrophage infiltration was significantly higher (*p* < 0.05) in tracheas infected with both Mass IBV isolates compared to the tracheas of uninfected controls (Fig. 6a and c). Similarly, lungs infected with either Mass IBV isolates had significantly higher number of macrophages (*p* < 0.01) than lungs from uninfected controls (Fig. 7a and c). In both the trachea and lung, there was no significant difference in IBV positive macrophages between two Mass IBV isolates (Figs. 6d and 7d).

## Discussion

The work described in this manuscript focused, first, to see the genetic differences of two Mass variant IBV isolates originating from layer flocks in two provinces of Western Canada. Second, we evaluated if infection of SPF chicken with genetically different Mass IBV variants would result in a different pathogenicity, tissue distribution and macrophage responses in the respiratory tract. Since the S1 protein is the main inducer of virus-neutralizing antibodies [52–54], either partial [31–33] or full-length [34–39] S1 glycoprotein gene have been used in many studies involving IBV molecular characterization throughout the world including previous Canadian studies. Using whole genome sequencing it has been shown that genes other than S1 may be playing roles in the pathogenicity of IBV [41, 55]. To our knowledge this is the first study, which investigated the full genome of Canadian IBV isolates. Although, IBV strains circulating in other countries have been characterized and changes in the IBV genome due to mutations and recombination have been shown [40, 55–59], our whole genome sequencing data shows that mutations are more common than the recombination and genes other than S1 have been affected by mutations. These changes in the genome of these two IBV isolates reflected in the differences in the pathogenicity although host response characterized by macrophage recruitment into the respiratory tract were similar.

Previously, studies have been conducted to compare pathogenicity and immune responses between nephropathogenic (i.e. QX like IBV strains) and Mass type IBV isolates recovered from various geographical areas [18, 42, 43, 45, 46]. Nephropathogenic QX-type strains are distantly related to the M41 strain and their genetic similarity is 78.6% [60]. Based on the partial S1 sequence analysis of three nephropathogenic IBV isolates, which belong to the Mass genotype revealed that they are more than 99.1 and 97.8% identical to the classical M41 strain in nucleotide and deduced amino acid sequences, respectively [61]. Since it is well known that very minor changes in the genome of viruses [47] including IBV [49, 62] lead to differences in pathogenicity and host responses, we chose two Mass type IBV isolates expected to have minor genome differences for evaluation of pathogenicity, tissue distribution and host responses. Mass type IBV isolates have been shown to replicate in the respiratory tissues, kidney, cecal tonsils and gastrointestinal tissues [18, 42, 43]. In agreement with these studies, we also observed that Mass IBV isolates originating from Western Canadian layer flocks replicate in tissues other than respiratory tissues. In our study, Mass IBV isolate originating in SK had significantly higher genome load in the lungs than the one originating in AB, though there was no difference of lung lesion score between these two IBV isolates. On the other hand, in trachea, the genome loads of these two Mass IBV isolates were not different, although the Mass IBV isolate originated in AB had significantly higher lesion scores. It is also noteworthy that Mass IBV isolate originating in SK was not detectable at 12 dpi in cloacal swabs. Since the major source of IBV genome in cloaca is due to IBV replication in gastrointestinal tract [63], it is possible that 15SK-02 IBV infection in gastrointestinal tract may have been cleared by 12 dpi. Overall, these two Mass IBV variants have similar pathogenicity with only slight differences which also agree with the minor genetic differences of these 2 Mass type IBV isolates.

Literature that describe pathogenicity using Canadian IBV isolates are scarce [8] Although this study evaluated the pathogenicity of 5 IBV isolates originated in Ontario poultry flocks, IBV shedding, tissue distribution and macrophage response have not been studied. Molecular characterization of IBV strains circulating in Canada has been done previously and it appears that there is a difference in distribution of IBV genotypes according to the geographic area within Canada [33, 37, 50, 51]. In Western Canada, mostly Mass and Conn IBV genotypes are common and in Eastern Canada, in addition to Mass and Conn genotypes nepharopathogenic strains, 4/91, CA1737 and DMV genotypes are common among poultry flocks.

It appears that host responses, characterized by respiratory macrophages following IBV infection is dependent on the IBV strain. For example, it has been shown that M41 but not Australian T strain increases macrophages in respiratory tract lavage [64]. Previously, it has also been shown that IBV ConnA5968 strain elicit higher macrophage response in tracheal mucosa when compared IBV M41 strain [65]. Although Mass IBV (M41) strain is known to increase macrophage responses in lungs and trachea [65], it is unknown whether variants of Mass IBV are capable of eliciting differential macrophage response in the respiratory mucosa. The current study shows that minor genetic differences in Mass IBV variants do not lead to differences in macrophage responses in the respiratory tract although each of the tested IBV isolates were able to elicit significant macrophage responses in these tissues. IBV M41 and ConnA5968 strains have been shown to replicate in macrophages in vivo and in vitro [65–67]. The IBV Beaudette strain is able to infect the HD11 macrophage cell line [68]. Although we did not observe a difference in macrophage infection in vivo by both Mass variant IBV isolates used in our experiments, it will be important to establish if these field IBV strains also target macrophages located in the respiratory tract for its replication. We observed a higher quantity of IBV antigens in 15SK-02 IBV infected lungs when compared to 15AB-01 infected lungs and that difference was not reflected in the number of IBV infected macrophages. This discrepancy may be due to the fact that IBV infects cells other than macrophages. Previously, we observed that IBV infection in macrophage of the respiratory tract was about 3–4% [65].

In spite of the novel aspect of our work comparing two Mass type IBV isolates with minor genetic variations for pathogenicity and host responses, there were some limitations to this study. First, we did not include a control group infected with the standard M41 IBV strain in our experiment and this was due to the limited infrastructure availability at our animal facility. However, in a separate study, we investigated the M41 IBV induced macrophage recruitment to respiratory tract [65]. Second, for the quantification of IBV, we employed real-time PCR technique targeting the most conserved N gene of IBV [69–71]. Since qPCR quantify both live and dead virus [72], our IBV genome load data do not reflect IBV replication. Alternatively, we would have determined viral titers using 9–11 day old SPF embryonated chicken eggs and expressed as EID_50_ [73]. Although this later method could be employed to determine IBV infectivity in our swabs and tissues, the use of this technique was precluded for quantifying IBV titer due to the requirement of large numbre of eggs.

## Conclusion

In conclusion, Mass IBV variants recovered from layer flocks had identical nucleotide sequence at ORFs 3a, 3b, E, M, 5a and 5b. The remainder of the ORFs showed 99.9% nucleotide similarity. Although, the genetic changes were minor, the pathogenicity in vivo of the Mass IBV variant originating from AB was different to the one that originated in SK, whereby the AB variant caused significantly higher tracheal lesions and replicated to a lower titer in the lung and lower genome loads in cecal tonsils. Additionally, both Mass IBV variants elicited host responses characterized by significant macrophage recruitment to the respiratory tract and there was evidence that both Mass IBV variants replicated within macrophages in the respiratory tract. Studies are underway to evaluate the pathogenicity of these two Mass type IBV isolates in laying hens.

## Methods

### Animals

Day old white leghorn (SPF) layer chickens (White Leghorn) were obtained from the Canadian Food Inspection Agency (CFIA), Ottawa, ON, Canada and housed in high containment poultry isolators at the Prion- Virology Animal Facility of the University of Calgary’s Foothills Campus with ad libitum access to feed and water.

### Virus

Mass IBV isolates originating from layer flocks in AB (15AB-01) and SK (15SK-02) were used in the study. Viral titers were determined using 9-day old SPF embryonated chicken eggs and expressed as 50% embryo infectious dose (EID_50_) according to the method described by Reed and Muench [73].

### Experimental design

#### Whole genome sequencing of mass IBV isolates that originated from Western Canada

The Mass-type IBV isolates from the current study were sequentially passaged in 9 to 11-day old embryonated eggs and allantoic fluid was harvested and concentrated using ultracentrifugation at 50,000 g for 3 h. The resulting pellet was fractionated using the Optiprep™ kit (Millipore-Sigma, Missouri, USA). Ribonucleic acid (RNA) was extracted from the sixth fraction and allantoic fluid directly. cDNA was synthesized, and quantitative polymerase chain reaction (PCR) assay was performed to quantify the IBV genome load.

#### Infection of chickens with mass IBV isolates originated from Western Canada

Two groups of 6 day-old unsexed-SPF chickens (*n* = 7 per group) were infected with 50 μL inoculum containing 2.75× 10^4^ PFUs of IBV isolates 15AB-01 and 15SK-02 intra-tracheally under isoflurane anesthesia. Another 5 of 6-day old SPF chickens were kept as uninfected controls. The chickens were weighed at 0, 2, 3, 4, 10, 12 and 14 dpi. At 2, 3, 10 and 12 dpi, oropharyngeal and cloacal swabs were obtained using Puritan® Unitranz-RT transport system (Puritan Medical Products LLC, Maine, USA) and stored at − 80 °C until further processing. At 4 dpi, 4 chickens from each infected and 3 chickens from the uninfected group were euthanized and trachea, lung, kidneys, cecal tonsils and reproductive tract samples were collected into RNASave® (Biological Industries, Beit Haemek, Israel) and stored at -20 °C. In addition, a portion of trachea, lung, kidneys, cecal tonsils and reproductive tract samples were collected into 10% neutral buffered formalin for histopathological examination and in Optimum Cutting Medium (OCT, Leica Biosystems, Wetzlar, Germany) for immunostaining. The remainder of chickens were continued to be observed and tissue samples were collected in RNASave® for genome load quantification at 14 dpi.

For the sampling of tissues, the chickens were euthanized using overdose of isoflurane anesthesia followed by cervical dislocation.

### Techniques

#### Sample preparation for whole genome sequencing

The virus fractions, which gave the highest genome load or the lowest Ct were selected from both isolates and cDNA concentration was determined using the Qubit 4 Fluorometer (Thermo Fisher Scientific, MA, USA). A total of 10–12 ng of cDNA from each isolate was sent for whole genome sequencing to the Swine and Poultry Infectious Diseases Research Center (CRIPA), University of Montreal, Canada where library preparation and sequencing performed on Illumina® Miseq (San Diego, California, United States) next generation sequencing (NGS) platform generating 150 bp paired-end reads.

#### Histology

Formalin-fixed terminal tissue samples were processed at the Diagnostic Services Unit of the University of Calgary’s Faculty of Veterinary Medicine including staining of sections with stained hematoxylin and eosin (H&E). Sections were examined under the light microscope and photomicrographs were taken under 40x magnification. The histological changes observed in the trachea [8] and lungs [69] were scored as described previously.

#### RNA extraction and cDNA synthesis

Tracheal and cloacal swab samples, which were in the transport media were vortexed and medium was transferred to 1.5 mL centrifuge tubes and centrifuged at 1000 g for 20 mins at − 4 °C. A total of 250 μL of supernatant was used for RNA extraction using the Trizol LS® reagent (Ambion, Invitrogen Canada Inc., Burlington, ON, Canada) following manufacturer’s instructions. Approximately 50 mg piece of tissue preserved in RNA Save® was homogenized in 1 mL of Trizol® reagent (Ambion, Invitrogen Canada Inc., Burlington, ON, Canada) using a Pro200 Power homogenizer (Diamed, Mississauga, ON, Canada) on ice and RNA was extracted following the manufacturer’s protocol. The RNA pellet was re-suspended in 20 μL of RNase-free water and quantified using the Nanodrop 1000 spectrophotometer at 260 nm wavelength (Thermo Scientific, Wilmington, DE, USA). A total of 2000 ng of RNA was transcribed using 10X RT random primers (High Capacity cDNA Reverse Transcription Kit, Invitrogen Life Technologies, Carlsbad, CA, USA) as has been instructed by the manufacturer.

#### IBV genome load quantification

The IBV genome load quantification was carried out following the products and protocol as described previously [65]. Briefly, real-time PCR method was used for IBV genome load quantification using forward primer, IBV N Fw: 5’GACGGAGGACCTGATGGTAA3’ and reverse primer, IBV-N Re: 5’CCCTTCTTCTGCTGATCCTG3’. Thermal cycling conditions were 95 °C for 20 s; 40 cycles of amplification/extension at 95 °C for 3 s, and 60 °C for 30 s followed by a melting curve analysis was done between 65 °C to 95 °C with an increment of 0.5 °C at every 5 s. Fluorescent acquisition was done at 60 °C for 30 s.

#### Immunofluorescent assay

Frozen tissues in OCT blocks were sectioned at 5 μm thickness using a cryotome (Leica Biosystems, Richmond, Illinois, USA) and adhered on to positively charged slides. The sections were air dried on the bench for 20 min, fixed by immersing in ice-cold acetone for 5 min and stained for macrophage and IBV antigens sequentially as has been described previously [65]. Briefly, after protein blocking with 2.5% horse serum, the cryosections were incubated for 30 min with mouse monoclonal anti-chicken macrophage (KUL01) antibodies (Southern Biotech, Birmingham, Alabama, USA) (1:200 in PBS containing 2.5% horse serum). The staining specificity of KUL01 anti-chicken macrophage antibody was confirmed previously using an appropriate isotype control (Additional file 1: Figure 1). The secondary antibody, DyLight® 550 conjugated goat anti-mouse IgG (H + L) (Vector Laboratories, Inc., Burlingame, California, USA) (1:500 in PBS containing 2.5% horse serum) was used. Before being stained for IBV antigen using anti-IBV rabbit polyclonal serum (Federal Research Institute for Animal Health, Greifswald-Insel Riems, Germany; 1:3000 diluted in 2.5% horse serum), the sections were blocked again with 2.5% of horse serum. For subsequent staining, Vectaflour RTU Antibody kit containing Dylight 488 conjugate (DK-1488, Vector Laboratories, Burlingame, California, USA). The sections were mounted on Vectashield® antifade mounting medium with 4′,6-Diamidine-2′-phenylindole dihydrochloride (DAPI) nuclear stain (Vector Laboratories, Burlingame, California, USA).

### Data analyses

#### Whole genome sequencing data analysis

All reads were assembled to get a single contig. The resulting full genome sequences were aligned, and identity matrix was performed with 43 published sequences of IBV reference serotypes retrieved from the National Center for Biotechnology Information (NCBI) GenBank database using the BLAST® research tool and the Custal Omega 1.2.3 package with automated settings (Geneious® 10.1.3). The phylogenetic tree inferred based on the full-length IBV genomes using Randomized Axelerated Maximum Likelihood (RAxML 8.2.7) method with GTR GAMMA nucleotide substitution model with 1000 bootstrap iterations (Geneious software, version 10.1.3, Biomatters Ltd., Auckland, New Zealand). The resulting proportional tree of the two Mass IBV isolates of this study with 43 reference sequences (Additional file 2: Table S1) is shown with the bootstrap support. Nucleotide sequences of the two Mass IBV isolates, 15AB-01 and 15SK-02 were deposited in the NCBI GenBank public domain under the accession numbers MH539771 and MH539772, respectively. ORFs of the two new sequences were determined using the NCBI ORF finder (https://www.ncbi.nlm.nih.gov/orffinder/) and the genome annotation was carried out using Geneious software, version 10.1.3 (Biomatters Ltd., Auckland, New Zealand).

#### IBV genome load quantification

IBV genome copies per 200 ng of cDNA were calculated using a standard curve based on a serial dilution of plasmids as described previously [69].

#### Quantification of the number of macrophages, IBV antigen and IBV positive macrophages

For the quantification of macrophages and the IBV antigen in the trachea and lungs, five areas with the highest DyLight® 550 (macrophages) and DyLight® 488 (IBV antigen) fluorescent signals and corresponding nuclear stained (DAPI) areas were captured under 40x magnification using an epifluorescent microscope (Olympus IX51, Center Valley, Pensylvania, USA). The obtained images were transferred to the Image-J® software (National Institute of Health, Bethesda, Maryland, USA) and the areas of each micrograph covered with DyLight® 550 (macrophages), DyLight® 488 (IBV) signals, combined DyLight® 550 and DyLight® 488 signals (IBV positive macrophages) and the combined DyLight® 550 and DyLight® 488 signals with DAPI (total area of the section) were quantified. The areas covered with macrophage and IBV antigens and the combined signals were expressed as a percentage of the total area covered by the section.

#### Statistical analyses

Two-way analysis of variance (ANOVA) followed by Benferroni post-hoc test was used to determine the significant time points of body weight among the infected and control groups. Tracheal and lung histopathological lesion score data was analyzed using Kruskal-Wallis test followed by Dunn’s multiple comparison test. The genome load data was analyzed by the two-way ANOVA followed by Bonferroni post-test. One-way ANOVA test followed by Tukey’s posttest was used to determine the significance of immunofluorescence data. Grubbs’ outlier test was performed in order to identify outliers before the data was analyzed. All the statistical procedures were performed using in-built statistical analysis feature of the GraphPad™ Prism 5 (GraphPad Software, La Jolla, California, USA). Differences were considered significant at *p* < 0.05. The significance on graphs are labelled as the following: *p < 0.05, **p < 0.01, ***p < 0.001, and ****p < 0.0001.

## Additional files

Additional file 1: **Figure S1.** Specificity of avian macrophage staining. The power point slide contain lung sections stained with anti-chicken macrophage antibody (KUL-01) and isotype control. The sections were also stained with nuclear staining (DAPI). (DOCX 271 kb).

Additional file 2: **Table S1.** List of reference IBV sequences used in this study. This table contain names and accession numbers of IBV whole genome reference sequences retrieved from the GenBank repository. (DOCX 15 kb).
