# Supplementary material for: Comparative features of infections of two Massachusetts (Mass) infectious bronchitis virus (IBV) variants isolated from Western Canadian layer flocks
Source: BMC Vet Res. 2018 Dec 10;14:391. doi: 10.1186/s12917-018-1720-9 (PMC6288874; doi:10.1186/s12917-018-1720-9)
Supplement: Supplementary file 1 — Figure S1. Specificity of avian macrophage staining. The power point slide contain lung sections stained with anti-chicken macrophage antibody (KUL-01) and isotype control. The sections were also stained with nuclear staining (DAPI). (DOCX 271 kb). [file 12917_2018_1720_MOESM1_ESM.docx]

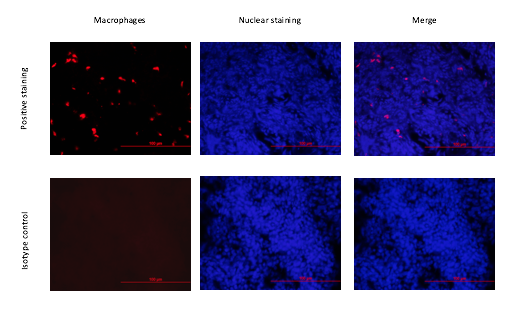


**Additional file: Figure S1:** Specificity of avian macrophage staining. Representative images of lung sections that were stained with anti-chicken macrophage antibody (KUL-01) or isotype control. The sections were also stained with nuclear staining (DAPI).
