# Supplementary material for: Comparative features of infections of two Massachusetts (Mass) infectious bronchitis virus (IBV) variants isolated from Western Canadian layer flocks
Source: BMC Vet Res. 2018 Dec 10;14:391. doi: 10.1186/s12917-018-1720-9 (PMC6288874; doi:10.1186/s12917-018-1720-9)
Supplement: Supplementary file 2 — Table S1. List of reference IBV sequences used in this study. This table contain names and accession numbers of IBV whole genome reference sequences retrieved from the GenBank repository. (DOCX 15 kb). [file 12917_2018_1720_MOESM2_ESM.docx]

| **Additional file: Table S1**: List of reference IBV sequences used in this study | | |
| --- | --- | --- |
|  |  |  |
| **Reference sequence** | **GenBank Accession #** | **Country of origin** |
| Strain 1148-A | KY933089 | United Kingdom |
| GammaCoV | KY620116 | China |
| Isolate VicS-del | KF931628 | Australia |
| 4/91 vaccine | KF377577 | China |
| QX type isolate AR251-15 | KX272465 | Sudan |
| IBV | KC008600 | China |
| NGA/A116E7/2006 | FN430415 | West Africa |
| Strain B1648 | KR231009 | Belgium |
| Isolate IBV-EP3 | DQ001338 | Singapore |
| Strain Beaudette CK | AJ311317 | United Kingdom |
| Isolate TN92-03 (India) | KR902510 | India |
| Mass41 1972 (USA) | FJ904721 | United States |
| Strain Mass 41 | AY851295 | United States |
| Strain ck/CH/LHLJ/091205 | KJ425504 | China |
| Strain M41 | GQ504724 | United States |
| Mass41 1965 | FJ904720 | United States |
| Strain GX- Mass41 (1985) | FJ904723 | United States |
| Strain H52 | EU817497 | China |
| Mass41 Vaccine | GQ504725 | United States |
| Strain Ma5 | KY626045 | Brazil |
| Conn46 | FJ904716 | United States |
| Mass/1009/13A/2015 | KY588135 | Pakistan |
| Isolate B17 | KT203557 | India |
| Strain H120 | FJ888351 | China |
| Isolate B17 | KT203557 | India |
| Serotype H120 | GU393335 | Unites States |
| Strain H120 (China) 2 | FJ807652 | China |
| Isolate ZJ971 | EU714028 | China |
| Isolate KM91 | JQ977698 | Korea |
| Strain SNU-9106 | KU900741 | Korea |
| Strain QIA-03342 | KU900739 | Korea |
| Strain QIA-Q43 | KU900744 | Korea |
| Serotype Holte | GU393336 | United States |
| Serotype Iowa 97 | GU393337 | United States |
| Serotype Gray | GU393334 | United States |
| Serotype JMK | GU393338 | United States |
| Arkansas vaccine | GQ504721 | United States |
| Serotype California 99 | AY514485 | United States |
| Serotype Cal56b | GU393331 | United States |
| Strain Mass41 2006 | FJ904713 | United States |
| Serotype FL18288 | GU393333 | United States |
| Connecticut vaccine | KF696629 | China |
| Strain Conn46 1972 (USA) | FJ904717 | United States |
|  |  |  |
